# Ten simple rules for supporting a temporary online pivot in higher education

**DOI:** 10.1371/journal.pcbi.1008242

**Published:** 2020-10-01

**Authors:** Emily Nordmann, Chiara Horlin, Jacqui Hutchison, Jo-Anne Murray, Louise Robson, Michael K. Seery, Jill R. D. MacKay

**Affiliations:** 1 University of Glasgow, Glasgow, United Kingdom; 2 University of Aberdeen, Aberdeen, United Kingdom; 3 University of Sheffield, Sheffield, United Kingdom; 4 University of Edinburgh, Edinburgh, United Kingdom; Carnegie Mellon University, UNITED STATES

## Abstract

As continued COVID-19 disruption looks likely across the world, perhaps until 2021, contingency plans are evolving in case of further disruption in the 2020–2021 academic year. This includes delivering face-to-face programs fully online for at least part of the upcoming academic year for new and continuing cohorts. This temporary pivot will necessitate distance teaching and learning across almost every conceivable pedagogy, from fundamental degrees to professionally accredited ones. Each institution, program, and course will have its own myriad of individualized needs; however, there is a common question that unites us all: how do we provide teaching and assessment to students in a manner that is accessible, fair, equitable, and provides the best learning whilst acknowledging the temporary nature of the pivot? No “one size fits all” solution exists, and many of the choices that need to be made will be far from simple; however, this paper provides a starting point and basic principles to facilitate discussions taking place around the globe by balancing what we know from the pedagogy of online learning with the practicalities imposed by this crisis and any future crises.

## Introduction

Coronavirus disease 2019, more commonly known as COVID-19, has acted as a severe disruptor for communities across the globe, not least for educational institutions. In the immediate response to COVID-19, many educational institutions internationally responded with an emergency “pivot,” in the sense of a change in mechanism, in their teaching, with many voluntarily or compulsorily closing their campuses for the final part of the 2019–2020 academic year. This pivot to online teaching has encompassed traditional on-campus programs delivering teaching and assessments online at a distance, many for the first time. The continuing impact of COVID-19 is different across the world, both in terms of the spread of the virus and the political response, and this has direct consequences for higher education. Institutions in China are progressing with plans to return to face-to-face teaching [1]; in the United States, Yale and Harvard have announced that the fall semester will be conducted online [2], whilst in the United Kingdom and European Union, many institutions have announced plans for blended learning, with additional global uncertainty created by the potential for second waves or local outbreaks. Plans for a temporary move to greater online delivery, whether fully remote or blended, will necessitate distance teaching and learning across almost every conceivable pedagogy, from fundamental degrees to professionally accredited ones. Each institution, program, and course will have its own myriad of individual needs (and each constrained by country-specific regulations, quality assurance procedures, and COVID-19 restrictions); however, there is a common question that unites us all: how do we provide teaching and assessment to students in a manner that is accessible, fair, equitable, and provides the best learning whilst acknowledging the temporary nature of this teaching? We must learn from and share best practices across the sector and not dismiss the findings in other disciplines, while recognizing that no “one size fits all” solution exists. The level of support, resources, and expertise will vary hugely between institutions, and many of the choices that need to be made will be far from simple; however, this paper identifies 10 guiding principles that balance pedagogical best practices of online learning with pragmatism necessary during this crisis. More than that, we aim to identify inclusive practices and approaches that can not only be used in any future emergency that would make on-campus teaching difficult but that may change higher education for the better as part of the new normal.

## Rule 1: A temporary pivot is not the same as emergency remote teaching or online distance learning

A temporary pivot to online teaching needs to be selective in what it incorporates from both emergency remote teaching and specialized online courses. Additionally, higher education (HE) management and policy makers must recognize the unique and fundamentally imperfect nature of this work and treat course evaluations, student satisfaction, and teaching quality assessments with extreme caution during this period. There is a fundamental and important distinction between this temporary distance teaching and what we traditionally consider online distance learning (ODL) via institutions like the Open University. Full ODL provision [3] or online training is neither new [4,5] nor a compromise in educational standards and is well received by students [6]. However, it is important to recognize that such research relates to students and staff who have chosen to teach and study online. For a proportion of the education community, teaching ODL courses is unfamiliar and likely to provoke anxieties about their in-person teaching being permanently replaced by online content. The Manifesto for Online Teaching [7] highlights some of the commonalities of online courses, be they open and free or fee-heavy and institutionally accredited. They are commonly developed with socioconstructivist pedagogies at their core (i.e., that knowledge is constructed through interaction with others as opposed to copied from a text or teacher), with the common mantra “distance is a positive principle not a deficit.” Accordingly, there is a strong focus on the online culture [8], with high production standards and the desire to widen access to education to a more heterogeneous studentship [9]. Many HE institutions involved in ODL have engaged with this at postgraduate levels, with only a few (such as the Open University in the UK and Athabasca University in Canada) entering into undergraduate provision. While many traditional on-campus HE courses may share these principles, few will have been designed from the ground up to embody them, and some educators see online resources as devaluing the classroom space [10].

There is also a further distinction to be made between the immediate reaction to the COVID-19 disruption, described as emergency remote teaching [11], and longer-term, but crucially still temporary, plans to continue teaching online. Programs of study with large practical components may never have voluntarily chosen to teach online and now need to consider what can and must be taught remotely. Those programs that adhere to professional standards, such as medical or engineering degrees, may need to consider how they can meet accreditation standards. For example, in the UK, Day One Competencies for veterinary degrees need to be met to allow for registration with the Royal College of Veterinary Surgeons (RCVS), and the General Medical Council requires all providers to meet the standards set out in the Promoting Excellence framework. Liaising with these bodies is important, as some aspects may be relaxed, e.g., the RCVS will allow for a small shortfall in practical experience in 2020 graduates. Contingency plans to deliver part of the 2020–2021 academic year fully online must be more robust than emergency remote teaching yet mindful that, for many, this is not the beginning of a brave new era but rather a challenge that must be met to balance workload, pedagogy, and practicalities until life can return to (a new) “normal.” It is also important to recognize that for some students, the best choice may be to defer studies for a year, and institutions will need to support and streamline the processes and procedures involved in these decisions. Quality assurance procedures will also need to be made more flexible to allow, for example, changing assessments and course structures.

## Rule 2: Provide asynchronous content

Unless interaction is necessary (see Rule 3), content that is recorded and provided asynchronously, so that students can access it at a time that is suitable for them, will allow students to engage with their studies in a flexible way. The Open University in the UK and similar institutions have extensive experience in distance teaching, and the Open University highlights particularly how flexibility is built into their teaching model. The move to online will hit disadvantaged students hardest. Barriers to education that may be further compounded for disadvantaged students during this time include a lack of access to quiet working environments, reliable access to technology, competing with other members of the household for access to that technology and/or data, and potentially underdeveloped skills necessary for independent online study [12,13]. This will be even further exacerbated for new first year students who have not had the opportunity to transition into “normal” university life, as well as those with caring responsibilities, mature students returning to study, and the many students who may be in different time zones across the world. It is therefore imperative that flexibility is built into blended learning, and academics must accept that we will have less control over where and when our students engage with our courses. Access to internet and digital resources will not be equal [14,15].

Additionally, just as traditional on-campus teaching began a gradual shift away from the notion of a prototypical “implied student” [16] and is being encouraged to embrace a studentship that is best supported by recognizing its diverse learning needs [17], so too is the (temporary) online student best supported with multiple opportunities and methods of engagement with core material at a flexible pace. The online learning environment is often a better environment for students that may not receive the necessary accommodations or have the flexibility they require to succeed in on-campus programs [18,19]. Similarly, online platforms provide an opportunity to reconceive and potentially optimize our methods of teaching to a heterogenous studentship by reconceptualizing traditional static methods (the 50-minute lecture). This inherently demands of us a self-reflection and empathic recognition of how our current teaching best serves students with the additional demands of increased self-management required to learn online and the lure of becoming “passive” or disconnected within a global climate of heightened anxiety and mental health vulnerability.

Best practice guidance for online courses recommends the repackaging of content to more discrete packages than we may be used to in typical face-to-face courses. One standard format includes videos of a suggested length of approximately 15 minutes [20] that are then able to be reviewed and are ideally tied to formative or summative assessment opportunities. However, in many cases, it may not be practical to redesign a lecture course to fit into these time limits as part of a temporary pivot. When reviewing how best to repackage more traditionally delivered content, consider the following:

- What content from a standard lecture can/should *only* be delivered by a lecture format?
- What content is foundational/background knowledge or revision that might be better shifted to self-paced pre-reading or other activities?
- What content might be better consolidated as post-lecture readings or extension materials, self-paced formative activities, low-stakes summative assessments, peer-to-peer small-group discussions, or facilitated seminars and Q & A sessions.
- For content that can and should only be presented in lecture form, consider how best this may be broken up to balance overwhelming students with numerous small recordings, sacrificing engagement with extremely lengthy recording, and/or making long recording inaccessible to students with streaming/bandwidth limitations.
- If visual content on slides is not used for active demonstrations in a recording, some students may benefit from copies of slides and the option of an audio-only recording to minimize cognitive and technological demands.
- How long do you expect students to spend on each asynchronous activity? Be mindful that if activities are removed from the constraints of a timetabled class, it can be easy to overload students with content.

## Rule 3: Provide synchronous and asynchronous contact and communication

Students should be given multiple avenues for both synchronous (students and instructors must be online at the same time) and asynchronous (students can engage on their own schedule) contact with staff and peers, and the differing intended purpose of each mode of contact must be clear. The expectations of both staff and students regarding engagement and responsiveness across each platform must also be explicit. Developing and maintaining academic communities and relationships will be the greatest challenge of temporary online teaching. Whilst lecture content should be provided asynchronously to allow flexibility, this does not preclude scheduling synchronous contact to provide students with the following:

- Open or scaffolded opportunities to seek support or clarification with regards to course materials or assessments.
- Consolidation of course content via peer- or tutor-mediated discussions.
- Social and academic networking opportunities.
- Development of collaborative skills during small-group activities.

For synchronous contact, it is important to enact as much control over your online space as possible, and you should explore the security settings available on your streaming software to minimize disruption and consult with your learning technologist. There is an increasing awareness of the disruption that synchronous online events can face (e.g., “Zoom bombing” [21]), and the guidance and support on how to set up video conferencing to prevent this is rapidly expanding. Additionally, do not forget your local digital space when streaming. For example, when screen-sharing as part of a lecture, be mindful to shut down programs that may use pop-up messages and notifications such as Outlook, Slack, or Microsoft Teams, and consider using an incognito private browsing window if sharing your browser to prevent your search history from appearing. When planning synchronous contact, it is critical to consider carefully what can be achieved in the time available. Delivery of activities such as active learning in face-to-face teaching relies heavily on the ability to read the body language and facial expressions of students. This is much more difficult with online delivery—even if you can see the faces of students, it will be challenging to get the same sense of whether they are keeping up from the nonverbal cues available. It is highly likely that it will take longer to deliver an activity online, and this needs to be considered when deciding what content should be covered synchronously.

Where appropriate, synchronous events should be recorded and made available to view at a later date to allow students to engage flexibly, particularly in recognition of time zone differences that may make live participation difficult. It is crucial that students are informed in advance which synchronous events will be recorded and shared, in much the same way that students are informed if live lecture capture is taking place on campus through appropriate signage and communication, so that they can make informed choices about their participation and, e.g., whether to put on their video. This is particularly important in subjects where the discussion and interpretation of arguments may involve espousing controversial viewpoints, as students “try on” new opinions and staff offer provocations or in clinical training in which sensitive patient information may be discussed. These are important learning experiences which require the management of a safe learning environment.

Asynchronous technologies provide opportunities for instructor–student and student–student time-delayed collaboration; the most commonly used asynchronous technologies in distance learning are discussion boards within the virtual learning environment (VLE), e.g., Blackboard, Moodle, or Canvas, and email to communicate in a text-based format [22], and these approaches are also of real value in a temporary move to online teaching. Asynchronous discussions using discussions boards in the VLE can have multiple threads with several discussions and interactions progressing simultaneously. Some students appear to prefer asynchronous communication in distance learning since, in addition to the flexibility afforded by the “anytime, anywhere” mode, this approach to communicating gives the learners time to reflect and respond. Being able to have the time to reflect can reduce apprehension in those individuals that are more likely to withhold their ideas from fear of others not approving [23]. Thus, text-based asynchronous interactions have been reported to facilitate academic discourse, socialization, and community building [24] through thoughtful and extended engagements at the students’ convenience [25]. However, asynchronous communication can pose issues for some learners, whereby time-delayed responses may result in messages appearing out of context and less meaningful, especially if a student has moved on to another topic or task [22]. They can also lack immediacy, which can limit some students’ responses to other students’ and instructors’ comments [26].

More recently, technologies such as Slack and Microsoft Teams have sometimes inadvertently superseded VLE discussion boards in spite of their intention of having independent but parallel purposes. This may be a result of the familiarity, informality, and accessibility of these newer app-based platforms, and we must be mindful of managing expectations and specifying the discrete nature of different platforms whilst simultaneously being mindful of just how many channels of communication we are asking students and staff to use [27]. These multiple methods of communication should be streamlined to as few as possible, each ideally having a single purpose, and both student and staff expectations regarding engagement and responsiveness for each should be managed and outlined clearly. It is encouraged that instructors take a balanced approach between maintaining an active role in managing conversations if required and allowing students to naturally guide some conversations themselves (see Salmon [28] for in-depth resources on structuring and moderating online learning).

It is important to acknowledge that, regardless of the specific platform, one of the challenges with text-based communication, especially if used in isolation, is a reduced form of social presence due to a lack of nonverbal cues, such as facial expressions and gestures [29]. Although often preferred by some neurodivergent learners [19], this can result in reduced context and greater ambiguity, as the reader cannot access the sender’s emotions unless the sender explicitly expresses them. The presence of social cues, such as smiles and encouraging gestures, has been seen to enhance learning [30], and the absence of these can promote a less personal and friendly approach to distance learning [31]. However, studies have shown that a sense of presence can still be established in text-based communication through the use of emoticons as a replacement for nonverbal cues [32], and this is particularly true for neurodivergent individuals with social anxiety or communication-related challenges [33]. Importantly, well-developed interpersonal relationships can be formed online through text-based communication [34], and online forums can foster a feeling of presence and create an online learning community [35]. It is also important to avoid the pitfalls of the “digital natives” idea: no one generation has better digital skills or more ability to work in digital environments than any other [36]. Whilst many students will be familiar with using social media, they will still need to be supported to use university educational technology, initially in a low-risk environment (e.g., an induction session) to build those skills and encourage conversations before subject-specific sessions occur.

## Rule 4: Set and communicate clear expectations about engagement

For many students, this will be the first time they have had to learn in a fully online environment, and it is vital that your expectations for engagement are communicated clearly at the beginning of the course. For example, when do you expect students to have viewed lecture content or completed the reading for each week (mindful of Rule 2)? How often should they expect to participate in synchronous events? How long should they spend on asynchronous activities? What monitoring and progress checks are in place? These expectations will help provide students with a structure that allows them to form a routine and will also help with the implementation of Rule 6 by setting clear criteria for successful engagement. Clear signposting (Rule 9) of what is required is essential, alongside clear information on what are the “core” learning activities and what are “additional” learning resources. You must also consider what you know of your cohort. There is evidence to suggest that veterinary students, when given prearrival materials online, remain anxious about workload and engage with materials mainly outside of normal working hours [37]. New entrants to university in 2020–2021 will have limited opportunity to assess their workload in comparison to their peers and need exceptionally clear instruction on how to spend their “independent learning” time. In the UK, a full academic year is typically 120 credits, equivalent to approximately 1,200 notional hours of learning; the European Union specifies that 60 European Credits Transfer System (ECTS) Credits [38] represent 1,500–1,800 hours at an appropriate difficulty level, whilst in the USA, a typical 15-week course represents approximately 180 hours. These time estimates, of course, do not describe structured learning time; the majority of this work will be independent learning, and students will need support for how to work independently to develop their skills in those hours.

It is also crucial to provide clear expectations for staff engaging with online teaching. Communications regarding staff expectations should be clear and concrete and point towards sources of help such as learning technologists and academic development that can help support their engagement with students. For courses that already involve substantial use of the VLE and online content, it can be helpful to highlight that the pivot may be more accurately described as blended to online, rather than offline to online, and provide examples of learning technologies staff have already engaged with as part of their regular teaching. The pivot to online may blur the boundaries between teaching and student support, and workloads and expectations should reflect the fact that online teaching is not simply about the hours spent in front of a camera. With regards to staff engagement with students, whilst we need to provide support, online spaces can blur the private and public spheres and can lead to an “always on” culture where students develop unrealistic and unreasonable expectations. Provide students with clarity about your availability, including online office hours, how long they should expect to wait for an email response, and alternative sources of support, such as peer-led discussion forums or chat groups. Second, consider your broader online presence and digital footprint. Simple steps such as increasing the security settings on your personal social media can help provide a distinction between the two spheres; however, for a deeper review, the University of Edinburgh have developed a Digital Footprint Massive Open Online Course (MOOC) that provides advice and practical approaches for managing your online presence and digital footprint.

Finally, allow and encourage control of the online spaces that students have, as well as their online professionalism or “netiquette.” Provide them with resources such as the Digital Footprint MOOC noted above, security tips for your institution’s video conferencing platform of choice, and existing resources from career services and student support surrounding online professionalism. Much derisory commentary is written by academics about how students write emails [39]. If you have expectations about the tone and style of online communication students should adopt, make these expectations explicit at the beginning of the semester rather than leaving students to fail and you to get frustrated.

## Rule 5: Design appropriate assessments with clear expectations

It may be tempting during this time to consider that assessments can simply be put online with very little change to the format or guidance beyond that which is practically necessary. There is considerable evidence, however, that assessment and, importantly, the associated feedback [40,41] are important moderators of student behavior [42] and help to build feelings of community and belonging with a students’ chosen discipline [43,44]. Socioconstructivist views of assessment and feedback consider feedback as a dialogic process, supporting the student to engage meaningfully with the quality of their work [45] and the demands of their new field [46]. In this framing, assessment is an important engagement point that **is** a learning experience in itself [47,48], as opposed to something that is “done to” learners or being used to evaluate the learning [49].

It is also important to recognize that any assessment on an online pivoted course will likely be open book and so may need design principles rethought and any change in expectations clearly communicated. For open-book exams, even if the type of questions and broad structure of the exam remain the same as in previous years, the format allows for students to focus on displaying comprehension and evaluation [50,51]. Thus, marking criteria will require a shift in focus from the recall of facts to comprehension and evaluation, and this should be made clear from the beginning of the course with appropriate guidance provided. For multiple-choice questions (MCQs), you may wish to consider options such as questions with multiple correct options as well as taking advantage of VLE features such as the randomization of response options to prevent sharing of answer keys. MCQ scoring systems should also be reviewed—negative marking reduces guessing but can disadvantage risk-averse students, and alternatives such as elimination testing may be more suitable [52]. Subjects with a reliance on “free-hand” methods, such as mathematics, physics, chemistry, or diagrammatic work in gross anatomy and biology may be considering assessments that require students to print out materials and scan in the completed hard copy. Whilst this may solve a technological problem, it adds further pressure on disadvantaged students (see Rule 2), who will be less likely to have access to printing facilities. Can students submit a scan via camera phone, and how will they know the quality of the photograph or scan is adequate? Considering these possibilities will allow you to provide guidance ahead of time. The time burden of online assessments should also be carefully considered to ensure students have the opportunity to successfully access, engage, and complete them. As noted in Rule 3, instructors are likely to find that they can achieve less in the same time, and our worries about workload should be extended to our students.

However, this period also presents an opportunity for less-traditional forms of assessment that are more suited to the open-book environment. As a generative alternative to MCQs, platforms such as PeerWise [53] allow students to create their own MCQs and answer their peers’ submissions and can be graded based on participation or by the accuracy of their answers or quality of their questions. In lieu of essay exams, students could be presented with work with factual errors and asked to assess the errors and correct them, detailing what resources they used and why in recognition that academic literacy is a sign of successful engagement [54]. At times, it seems that many HE practices, such as closed-book exams and large didactic lectures, have survived because of tradition rather than their pedagogical worth. Being unable to do things the way we have always done them presents us with the greatest challenge teaching and learning in HE has collectively ever faced; however, it also presents us with an opportunity to take stock of what is important and create authentic assessments that test the skills we really value.

## Rule 6: Monitor and support engagement

For the benefit of both their education and their well-being, students’ engagement with the course, their peers, and their lecturers should be regular and sustained. Student engagement has been referred to as a “meta-construct” that consolidates various aspects of student attainment and satisfaction [55]. Engagement can be framed in terms of the students’ behavior (e.g., are they attending? Are they talking with friends in the lecture?), the psychological processes of learning that occurs within the engaged state, and the students’ assimilation into academic culture [56].

Throughout all framings, engagement is considered an extremely important aspect of student learning [57]. Consider how you will monitor behavioral engagement (e.g., how much material students have engaged with) and psychological engagement (e.g., their new identities as online learners and how they are coping with the transition). Whilst being mindful of the need for extra flexibility, it is important not to confuse flexible learning with unsupervised learning. For asynchronous content, engagement can be monitored through learning analytics or by asking students to self-report through features such as Moodle Checklists or Blackboard Tasks. For synchronous contact, given the additional demands and time zone differences many students will be facing, it would be inappropriate to penalize failure to attend; however, this does not mean that attendance should not be monitored and used to identify students who may have additional difficulties and pressures on their time who need extra support. During synchronous contact, it will likely be more difficult to detect students that are struggling or who are disengaged “lurkers” as one could in offline teaching. The use of regular comprehension and engagement checks through, e.g., online polls and MCQ questions may help instructors regain some of the knowledge that will be lost through a reduction in nonverbal cues. Above all, monitoring should be compassionate and conducted with the recognition that, for some, “getting through” the year may be a very great victory.

## Rule 7: Review the use and format of recorded content

Whilst the use of recordings from previous years may be appropriate, pivoted lectures may also need to be recorded anew to ensure suitability for online-only delivery. For example, there may be parts of the lecture that do not translate online (e.g., a discussion that isn’t picked up by the mic), technical issues such as microphone failure that have not been previously noted, reference to an assessment that no longer exists, or the use of an off-camera tool like a chalkboard. These issues may be relatively minor, but the impact of such incongruencies will be exacerbated for new students who have not experienced a lecture first-hand or as a supplement to a live event. Additionally, to help develop and maintain community, the use of old recordings should be accompanied by introductory videos in which the lecturer introduces themselves directly to the new cohort. These videos can be short, but they will help foster a connection between staff and students that risks being damaged if students are only provided with old content, particularly for new first-year students. Last year’s lectures may reduce workload compared to recording anew; however, they will still require time from learning technologists, if not necessarily from the lecturers themselves, and HE and Further Education (FE) providers need to consider this “invisible” time burden.

It is also important to ensure that the decision to reuse lecture recordings is in line with any existing lecture capture policy. Many institutions that have an opt-out policy [58] explicitly require permission from the lecturer to reuse a recording beyond the year and the course that it was initially recorded for. Whilst this is a very different scenario than most policy makers will have had in mind, it is important not to undermine the integrity of the lecture capture policy, and so explicit permission should be sought (arguably, regardless of what policy exists). This is particularly pertinent for studies of the arts, for which performance rights of staff and students may clash with copyright of certain pieces. Working with library services from an early stage can help avoid these pitfalls. If asking students to record pieces and submit for performance assessment and feedback, ensure they understand copyright and privacy implications of this practice.

Research on whether the visual presence of the instructor in instructional videos aids learning is mixed. Fiorella and Mayer [59] suggest that it does not greatly impact learning, as do Kizilcec and colleagues [60]; however, the latter also conclude that although social cues may not enhance learning per se, they may affect learners’ motivation to persist in a course. It is important to remember that the COVID-19 cohort will not have chosen to learn through video, and so any social impact is likely to be greater; therefore, we advise that, when possible, the instructor should be visible in recorded content. Even a still image of the lecturer can help give students a strong sense of community (see Rule 9) and may be a more reasonable change to make to content that already exists.

On a more practical note, fully online programs expend a great deal of time and effort to ensure that video output is high quality and that disfluencies, etc. are edited out of the final output, whilst video output during emergency remote teaching may have been by any means necessary by teachers still getting to grips with new technologies. Whilst lecturers may wish to top-and-tail their videos to avoid any deadtime or lecture setup from being captured, there should be no expectation that the video output for a temporary online work will be the same quality as the content of an online program. This is not to fantasize that the process of pivoting online will not take a great deal of work—it will—but rather to emphasize that efforts should be focused on building community and engagement rather than video editing.

Finally, consider using open educational resources (OERs) as an alternative to creating or supplementing new material. Terabytes of high-quality educational video and/or short online courses already exist via platforms such as YouTube and OpenLearn [61] and, in addition to reducing workload, may present students with a different perspective.

## Rule 8: Focus on achievable learning outcomes for field, laboratory, and performance

Disciplines that have a large practical component that are often stipulated by professional and accrediting bodies [62–64] require additional thought in moving online, especially if only temporarily. This includes field, laboratory, and performance work, which would normally occur in highly specialized and structured environments or as part of an ensemble. For science subjects, while online laboratories cannot replace the tangible aspects that are gained in person, there is substantial literature on the value of online laboratories for a range of learning outcomes [65], and lessons can be learned from institutions such as the Open University, which teach practical science remotely and have made help sheets available to the wider education community [61]. It is useful to structure the overall activity on aspects relating to experimental planning, decision-making, and reviewing real data—key attributes in typical professional accreditation activities and benchmark statements. In fact, these are activities that often do not appear in traditional laboratory instruction [66], and there is ongoing criticism of poor learning in laboratory environments [67,68]. Therefore, there is an advantage in including activities relating to experimental design and argumentation of data that are known to improve student outcomes in laboratory work [69] in an online context, as they are beneficial activities that do not actually require physical laboratory work. This approach can be augmented by simulations [70], virtual labs [71,72], or videos [73,74] that develop students’ understanding of technical processes relating to laboratory techniques so that the combination of laboratory technique and laboratory process provides an effective overall experience. Many of the strategies for teaching practical skills are also relevant in online environments, such as creating a “stress-free” safe learning environment and breaking the skill down into manageable, observable steps [75]. For arts and performance-based fields, the same principles regarding focusing on achievable learning outcomes apply. Organizations such as the Musicians’ Union have developed guidance for how to support online teaching [76]; however, the challenge for practice-based arts courses should not be underestimated or minimized compared to the discussion of science subjects. Video-conferencing technology does not yet allow for ensemble activities such as choirs or orchestras to take place due to issues with lag, and community groups have been faced with the challenge of continuing the pastoral and community elements in the absence of being able to perform together [77].

As with many aspects of (online) teaching, an especially important consideration is that of the instructor, as their influence heavily impacts the students’ affective experience of the online class [78], indicating that careful thought is needed in planning student support of online practical activities. Further, these practical skills most often rely on the student integrating feedback from peers and tutors with their own practice, and even if translated online, we should expect them to require a great deal of staff time. Where possible, it may be more feasible to delay practical work to the start of 2021, “front loading” degrees with theory, although this has the potential to “silo” learning and make the integration of knowledge and skills harder. This strategy may not be viable for longer, open-ended projects such as research projects or courses with a heavy performance component or that require access to artistic materials and equipment, especially if the second half of the academic year will feature more in-class activities than is typical. Any dissertation supervisor will be aware of the pitfalls of starting a dissertation project too late in the year. Practical activities on more open-ended topics, such as undergraduate research projects, could draw on citizen science initiatives for data sourcing and analysis [79], and accrediting bodies such as the British Psychological Society already allow for secondary data analysis and computational modelling for their empirical practical component [80,81]. In the longer term, there is benefit from considering how the use of online materials developed for the immediate post-lockdown response can be of value. There is a substantial literature on the use of simulations, videos, and other resources that allow students to prepare in advance for laboratory work [82], with many reporting virtual laboratory simulations to be equally as effective (if not better, in some cases) in preparing students for physical laboratory practicals [83]. Performance-related skills for artists such as the management of an online profile or the creation of digital content may also be credible alternatives; whilst this does not replace the practice-based learning that would take place, these are skills that students will find valuable in a post-COVID world. Thus, while preparing materials for a short-term response to distanced teaching, consideration for their longer-term value is worthwhile.

## Rule 9: Ensure resources are available, accessible, and signposted

Whilst much of the focus will be on pivoting lectures and labs online, it is important not to forget about associated resources. For example, ensure that material included on reading lists does not require physical access to the library. This may require the temporary use of less-favored alternative material; however, it is these small details that are likely to make a difference to student engagement and retention, and reading lists that contain material they cannot access will likely create a negative impression that may be difficult to overcome when “normal” service resumes. Many publishers have made textbooks and additional online resources available for free (see Jisc [84] for a maintained list of resources) to allow students access to study materials during COVID-19 disruption. How long this will continue is unknown, but publishing reps should be consulted to determine whether there are resources available to help support the pivot. Equally, academics should work with librarians who can provide support to identify online resources and suitable alternatives. This includes access to computing teaching resources, such as server time, specialist software, and datasets. For many programs, this may be a push towards more open science practices, whether that be exploring the value of open datasets [85,86], the use of open tools [87,88], frameworks such as the FAIR [89] principles, or even how OERs can facilitate programmatic work [90]. Not all programs of study will be able to adapt to fully open and online practices, and those that cannot must establish new ways of accessing limited resources, be that server time or physical spaces, when students return. Those that can must ensure that clear walkthroughs for the installation of software or virtual private networks (VPNs) are provided. When creating online content or posting already-created material online, it is important to ensure compliance with copyright law. Jisc provides a number of resources regarding digital copyright for UK institutions [91]; however, these rules are different globally (for example, in Sweden, teachers may retain copyright or make materials accessible via an Open Access license). Again, consultation with your institution’s librarians can provide support in how to interpret copyright and fair use policies and the relationship between performance rights and copyright within your own institution and country.

Alongside the provision of textbooks and papers, associated content such as PowerPoint slides to accompany lecture recordings should not be forgotten; these can help students structure notes without losing the message of the material being presented [92]. Many institutions will already have an accessibility-related policy of ensuring lecture slides are available in advance, in line with guidance from Jisc [93]; however, adherence to these policies can sometimes be patchy, and it is crucial that efforts are made to ensure compliance across all teaching. The most recent digital accessibility guidelines must also be taken into consideration [94]. Whilst much work has already been done to ensure the accessibility of existing online learning materials, it is vitally important that any new material follows these guidelines; otherwise, the pivot risks alienating and further disadvantaging disabled students. For example, for blind and visually impaired students, digital documents must be able to be read by a screen reader, and videos may need audio description to be accessible. For deaf and hard-of-hearing students, video content will need captions, and consideration should be given to synchronous online events such as whether an interpreter may be necessary or if transcripts can be shared afterwards.

Methodical organization of online spaces and signposting of resources will be paramount to reduce confusion and anxiety, particularly for new students yet to experience the VLE. Learning technologists will be of primary importance in providing support, and academics and administrative staff should work with learning technologists throughout all stages of the planning process. Short navigation videos will help to provide clear guidance and increase student confidence in finding their way around, in addition to platforms such as Microsoft Teams, which have the capacity to create classroom notebooks/classroom materials folders. It is likely that a variety of platforms for communication and content will be required; therefore, it will be vital that clear signposting is used at the beginning of term to advise students what platforms will be utilized for which purposes. Additionally, clear guidance must be provided on how to access/use these and a clear rationale provided for any tasks they are given: failure to do so will risk student engagement. Whilst the process of finding alternative, accessible materials and providing additional guidance via short video capture seems yet another pressure during a challenging time, this is an opportunity to future-proof courses and reassess the materials currently used with a view to their utility and accessibility. Incorporating these changes now will certainly have costs in terms of preparation time in the short term but may produce benefits for both staff and students not only during this temporary pivot but in the longer term.

## Rule 10: Create a community for staff and students

The success of the online pivot will not be determined by the quality of video content but by the strength of the community that emerges from the other side. Online communities can be very strong, particularly in education. The so-called “campus imaginary” can be a powerful draw for the distance learning student, where they feel part of a community that may not be entirely reflective of the on-campus experience [95]. Even classes of a thousand students can feel strongly bonded to lecturers with simple techniques such as welcome videos and question-and-answer sessions, especially when this comes at a difficult period in the student’s life [96]. It is likely that there will need to be increased communication, such as daily updates, particularly at the beginning of term. Even if there is no new information to be conveyed, until students (and staff) adapt to the pivot, consistent and transparent communication will be vital.

Many students entering their first year in a pivoted environment will have missed the big social events that mark their transition into a new life stage, e.g. prom, final year dance. They will be entering university without a traditional freshers’ week, or a freshers’ week that at the very least will carry new anxieties about a crowded room. These students are more likely to feel vulnerable and unhappy that their university experience is not the one they were looking forward to or the one that their older friends and siblings experienced. For many, this has the potential to be an extremely isolating experience, and therefore, creating and building a community will be vital to distort that feeling of distance and isolation. These students will also likely be experiencing university during an economic downturn and will have greater anxieties, either about their current or future circumstances. Students transitioning into an honors year or a practical course year will feel short-changed, as did students who missed out on fieldwork opportunities during the UK’s Foot and Mouth crisis [97]. These disappointments will also need to be monitored and supported. With students in such a vulnerable state and community so important, we should again strongly question the need for teaching quality assessments in this period. For example, in the UK, the National Student Survey is criticized for having vague questions [98], and asking students about their learning community in this time period is unlikely to lead to usable information. Students should also be supported in building their own peer communities (for example, by providing training in how they can use video conferencing to run study groups, peer-assisted learning schemes, or even virtual coffee) to help plug the gap for the many and varied social interactions that would normally play a large role in university life.

The need to maintain community also applies to staff. It cannot go unrecognized that for many academics, learning technology and online teaching is as alien as it is stressful, and some may have conceptualized teaching and learning as existing wholly within the lecture hall and find this transition challenging. Just as we must ensure there is no hidden curriculum for successful online learning for students, there can be no hidden curriculum for successful online teaching. Instructors need to be supported according to their level of expertise and plans to pivot teaching need to consider that expertise. Some may relish the opportunity to transform a traditional lecture course into a truly online experience; others should focus on small adaptations that, as per Rule 1, seek to make the best of an unprecedented situation. Clear leadership and communication from management that shows an understanding of these considerations, and minimum expectations will help staff at all levels of online experience. In addition to institutional support from academic development teams, resources such as the Quality Assurance Agencies Technology Enhanced Learning Hub [99], Developing a Sense of Belonging in Online Distance Learning [100], and How To Teach Online: Providing Continuity for Students [101] may be helpful to share with teaching teams.

All institutions need to consider the costs and benefits of introducing mixed cohorts in which some students attend on-campus and other students attend online. The on-campus experience will be strongly defined by the strength of social-distancing measures, which may impact timetabling such as running multiple lectures to accommodate class sizes. It may also be difficult to ensure equity of experience for online students, who will include international students and students with underlying health conditions. A blended model will also have an impact on staff time, with each piece of content necessitating repeated delivery, and will require presence on campus, which carries the same concerns as for students. Even if, as discussed above, lectures are reused, the contact time must be spread across two cohorts. The increased cost of staff time and the impact on class community may make blended models a challenge to successfully implement.

One unfortunate aspect of COVID-19 is that it has highlighted division within communities and countries, with partisanship seemingly influencing compliance to disease control measures [102–104]. Many HE providers responded to increasing globalization through a neoliberalization of education markets in the 1980s and 1990s [105], and now, the threat of a collapse in demand for international students greatly concerns the sector, even when that threat comes from government policies [106,107]. Individual HE providers will need to navigate their own local (in terms of their ties with their immediate towns and communities and the risks of bringing in large populations of students), national (in terms of their relationships with their own governments and auditing bodies, who often control large amounts of funding), and global (in terms of their international reputation and rankings) communities to successfully weather this crisis. We reiterate that “no one size fits all” here, but we make a plea for compassion. The community matters.

## Conclusion

Despite the difficulties and uncertainty that lie ahead, we should not lose sight of the potential for this crisis to result in lasting positive changes to higher education. Online learning demands that greater attention is paid to accessibility and inclusivity; our inability to stack students into a large exam hall at the end of term may be the driving force we need to design more authentic assessments, and much reflection has taken place over what truly matters when it comes to learning and what it is that makes universities exciting and challenging places to work and study. These are all good things. No guidance could possibly answer all the questions that will face each institution and program leaders over the coming months, and there will be many case-by-case decisions to be made, some of which will have no right answer. However, we hope that this paper provides a framework and some guiding principles upon which to base discussions that have community and inclusivity at their core. To co-opt Maya Angelou, students will forget what software we used, they will forget the mistakes we made in a time of crisis, but they will never forget how we made them feel.
